# Pulmonary Arteriovenous Malformations: A Rare Cause of Ischemic Stroke

**DOI:** 10.7759/cureus.5141

**Published:** 2019-07-15

**Authors:** Caleb J Heiberger, Mark J Brown, Divyajot Sandhu

**Affiliations:** 1 Radiology, University of South Dakota Sanford School of Medicine, Sioux Falls, USA; 2 Neurology, University of South Dakota Sanford School of Medicine, Sioux Falls, USA

**Keywords:** pulmonary ateriovenous malformation, may-thurner syndrome, ischemic stroke

## Abstract

A 24-year-old woman was admitted for seizures. Magnetic resonance imaging revealed a subacute infarct of the right frontal operculum. Transthoracic echocardiogram showed evidence of patent foramen ovale (PFO). Further study with transesophageal echocardiogram showed no PFO, but signs of a pulmonary arteriovenous malformation (PAVM) that was confirmed on ensuing chest CT angiogram. May-Thurner syndrome (MTS) was suspected and confirmed by magnetic resonance venography showing 70% narrowing of the left common iliac vein. The PAVM was successfully coiled and the patient was discharged without deficits. Noncontrast CT at one-month follow up showed no residual PAVM sac. Literature shows there is a median two-year delay from cerebral event to diagnosis of PAVM. Over 80% of PAVMs are related to hereditary hemorrhagic telangiectasia (HHT) and are generally seen in multiples, but may also been seen as an idiopathic and/or isolated defect. The risk of neurological complications rises with a patient’s age and the quantity of PAVMs. Initial workup should include screening with transthoracic contrast echocardiography followed by CT angiography for definitive diagnosis. Embolotherapy is considered gold standard as it reduces the risk of paradoxical emboli and other complications.

## Introduction

Pulmonary arteriovenous malformations are an often unconsidered etiology in evaluation of acute neurological complications as evident by the median two-year delay from cerebral event to diagnosis of PAVM [1]. Previously considered a rare disorder with an estimated incidence of 2-3 per 100,000, recent reports indicate a higher frequency of approximately 1 in 2,600 [1-2]. Acute neurological complications, commonly ischemic stroke and brain abscess, are the presenting complaint in 19%-59% of cases [3-4].

## Case presentation

A 24-year-old right-handed woman initially presented to an outlying facility by emergency medical services after being found covered in vomit and shaking on her bathroom floor. Glasgow Coma Scale upon arrival was 7 (E1, V2, M4) and she was intubated after 2 mg of intravenous lorazepam and 25 mg intravenous ketamine stopped her convulsions. Levetiracetam was given and an ensuing CT scan was read as having a right posterior frontotemporal 4.7 cm x 3.6 cm lesion with mass effect measured as 5 mm right to left shift. She was transferred by flight to our hospital.

**Figure 1:**
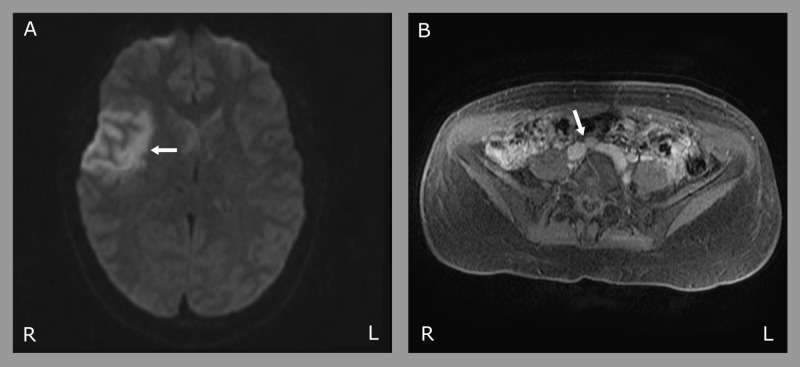
MR imaging. (A) Axial diffusion weighted imaging (DWI) MRI showing a subacute right frontal opercular infarct. (B) Axial pelvis magnetic resonance venography (MRV) showing compression of the left common iliac vein by the overlying right common iliac artery and the posterior vertebral body consistent with May-Thurner syndrome.

Magnetic resonance imaging revealed a subacute right frontal opercular infarct and an old cerebellar infarct (Figure 1A). Medical history was significant for smoking and levonorgestrel use as well as a family history of stroke at age 45 in her mother. Neurological exam was initially limited due to intubation. Magnetic resonance angiography (MRA) of head and neck was negative. Transthoracic echocardiogram revealed evidence of what was thought to be a large PFO. Venous ultrasonography of extremities and thrombophilia panel were both unrevealing. May-Thurner syndrome (MTS) was suspected and confirmed by magnetic resonance venography (MRV) showing 70% narrowing of the left common iliac vein by compression from the right iliac artery but no thrombus (Figure 1B).

**Figure 2:**
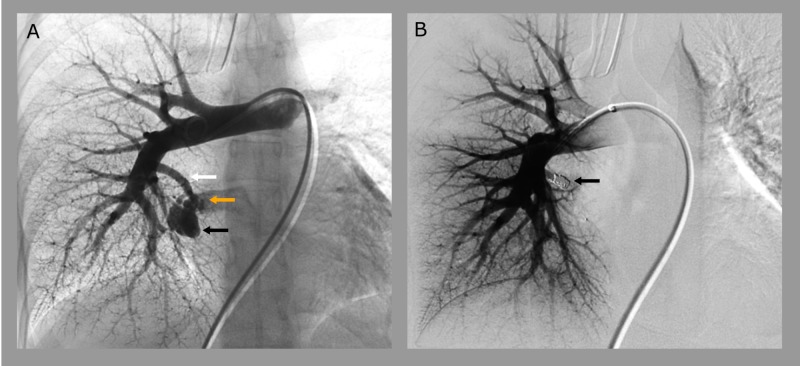
Coil embolization. (A) Digital subtraction angiogram (DSA) confirming a 1.6 cm x 1.3 cm x 1.5 cm pulmonary arteriovenous malformation (PAVM) located in the right lower lobe (black arrow) with a 4 mm feeding artery (white arrow) and a 7 mm dilated draining vein (orange arrow). (B) DSA highlighting complete occlusion of the PAVM with coil embolization of the feeding vessel (black arrow).

Following extubation, neurological exam showed no deficits. Transesophageal echocardiogram with bubble study to assess viability of PFO closure showed bubbles in the left atrium after five beats indicative of a PAVM. Subsequent chest computed tomography angiography (CTA) demonstrated a PAVM measuring 1.6 cm x 1.3 cm x 1.5 cm located in the right lower lobe with a 4 mm feeding artery and a 7 mm dilated draining vein. She was started on low intensity heparin drip. Interventional radiology was consulted and planned to perform AVM embolization with possible iliac stenting within the week. She remained stable and on fifth day of admission underwent a digital subtraction angiogram (DSA) confirming a PAVM as described on CTA (Figure 2A). The feeding vessel was successfully embolized, obtaining complete occlusion of the PAVM (Figure 2B). No thrombus was visualized and the patient was asymptomatic, therefore, stenting was deferred. Her levonorgestrel intrauterine device was removed and replaced with a copper device on the day of her embolization. The patient was discharged on 325 mg aspirin daily and 500 mg levetiracetam twice a day. Noncontrast CT at one-month follow up showed no residual PAVM sac and the patient continues to live unimpeded by complications.

## Discussion

Previously considered a rare disorder, recent reports show PAVM have a frequency of approximately 1 in 2,600 [1-2]. Acute neurological complications, commonly ischemic stroke and brain abscess, are the presenting complaint in 19%-59% of cases [3-4]. Despite their commonality, a median two-year delay exists from cerebral event to diagnosis of PAVM [1]. As in our patient, but less frequently overall, seizures can occur as well [3]. Over 80% are related to HHT and are generally seen in multiples, but may also been seen as an idiopathic and isolated defect [2]. There was no family history in our patient indicating HTT. The risk of neurological complications rises with a patient’s age and quantity of PAVMs with incidences of 10% below and 45% over age 30 and odds ratio of 4.5 comparing multiple to single lesions [5-6].

The strongest risk factors for ischemic stroke in the setting of PAVM are low serum iron, low pulmonary artery pressure, and high serum fibrinogen [1]. Iron deficiency and fibrogen excess promote thrombus formation and low pulmonary pressures facilitate the paradoxical embolism process [1]. In our case, MTS, a relatively common condition without reported association with PAVMs, was thought to be the source of emboli [7].

Initial workup should include screening with transthoracic contrast echocardiography followed by CTA for definitive diagnosis [1-2]. A feeding artery greater than 2-3 mm and paradoxical emboli are indications for intervention [2]. Embolotherapy is considered gold standard as it reduces the risk of paradoxical emboli and other complications [2]. Collateralization and recanalization may occur, thus life-long follow up is advised [4]. Our patient was successfully treated with coil embolization and remained asymptomatic at one-month follow up.

## Conclusions

Pulmonary arteriovenous malformations are an underappreciated cause of life-threatening neurological events. They should be suspected in individuals without evidence of more common etiologies. Successful intervention reduces the frequency and recurrence of neurological complications.
